# Verification and validation of an automated robot inspection cell for automotive body-in-white: a use case for the VALU3S ECSEL project

**DOI:** 10.12688/openreseurope.13627.1

**Published:** 2021-09-29

**Authors:** Alper Kanak, Salih Ergun, Ahmet Yazıcı, Metin Ozkan, Gürol Çokünlü, Uğur Yayan, Mustafa Karaca, Abdullah Taha Arslan

**Affiliations:** 1 Ergünler R&D Ltd. Co., İstanbul, Turkey; 2 Department of Computer Engineering, Eskisehir Osmangazi University, Eskisehir, Turkey; 3 Otokar A.Ş., Sakarya, Turkey; 4 İnovasyon Mühendislik TGD Ltd. Co., Eskişehir, Turkey; 5 Techy Ltd. Co., Eskisehir, Turkey

**Keywords:** cyber-physical systems, verification and validation, cyber-physical security, safety, privacy, automation, robotics, industrial quality control, vulnerability, resilience

## Abstract

Verification and validation (V&V) of systems, and system of systems, in an industrial context has never been as important as today. The recent developments in automated cyber-physical systems, digital twin environments, and Industry 4.0 applications require effective and comprehensive V&V mechanisms. Verification and Validation of Automated Systems' Safety and Security (VALU3S), a Horizon 2020 Electronic Components and Systems for European Leadership Joint Undertaking (ECSEL-JU) project started in May 2020, aims to create and evaluate a multi-domain V&V framework that facilitates evaluation of automated systems from component level to system level, with the aim of reducing the time and effort needed to evaluate these systems. VALU3S focuses on V&V for the requirements of safety, cybersecurity, and privacy (SCP). This paper mainly focuses on the elaboration of one of the 13 use cases of VALU3S to identify the SCP issues in an automated robot inspection cell that is being actively used for the quality control assessment of automotive body-in-white. The joint study here embarks on a collaborative approach that puts the V&V methods and workflows for the robotic arms safety trajectory planning and execution, fault injection techniques, cyber-physical security vulnerability assessment, anomaly detection, and SCP countermeasures required for remote control and inspection. The paper also presents cross-links with ECSEL-JU goals and the current advancements in the market and scientific and technological state-of-play.

## Plain language summary

Since automated systems are getting more complex, Industry 4.0 applications face a significant overhead on the verification and validation (V&V) processes making them time-consuming and costly. Verification and Validation of Automated Systems’ Safety and Security (VALU3S), a Horizon 2020 Electronic Components and Systems for European Leadership Joint Undertaking (ECSEL-JU) project started in May 2020, aims to design, implement and evaluate state-of-the-art V&V methods and tools in order to reduce the time and cost needed to verify and validate automated systems with respect to safety, cybersecurity, and privacy (SCP) requirements. This paper focuses on such problems with a special focus on applying V&V for improving manufacturing quality in the automotive sector. The targeted use case presents a novel approach to tackle the SCP requirements of an automated robot inspection cell for automotive body-in-white, and the underlying sophisticated techniques can be extended to a wider context for better V&V of the quality inspection procedures in complex environments.

## 1 Introduction

Quality control has never been important as today, especially in fabrication processes, as it reduces costs and increases the manufacturing efficiency and quality in complex factory environments. With the rise of Industry 4.0, smarter techniques used in quality inspection and control and the effective monitoring of fabrication processes facilitate the entire product life cycle. Here, trustworthiness in such fabrication processes plays a crucial role as there has been an increasing demand for the protection of workers and environments against safety problems, and the improvement of the resilience of infrastructures against cyber-physical security issues and privacy concerns. Aligned with this vision big industrial companies eventually invest in trusted quality control and management systems to keep and increase their reputation in the market ^1^.

A product that is lacking in quality control, especially in the automotive industry, can trigger expensive recalls. Vehicle components which are manufactured with low quality may even cause serious accidents and these accidents can be hazardous to drivers, people around the vehicle, and infrastructures across roads and their neighborhoods. Quality control can also spot problems before the product is marketed to consumers, leading to better releases. Quality inspection plays a crucial role in the automotive industry as it ensures that these products and the underlying components meet industry standards, e.g., the exhaust and emissions systems, wireless communication, security and safety.

As automotive technologies are getting more diversified, testing all components of a vehicle can become more complex and time-consuming. Additionally, safety is always a matter of particular concern not only at fabrication stage but also in actual use. In fabrication stage, the workers and technical teams are always in danger especially when working on product line operations, and while assembling or disassembling components, etc. In such cases, the main reason for an accident or abnormal behavior of a functioning unit can either be human-related or due to a cyber-physical system (e.g robotic components, machinery, etc.). There can be failures, intentional or unintentional damages, interception or intrusion attacks, or even internal or external nefarious activities that can cause serious defects and even lock-downs in fabrication processes. Safety concerns should be considered in long-term, even starting at the early phases of design. Secure-by-design, safety-by-design and privacy-by-design are key factors to prevent such damages and any undesirable situations that may even occur at the post-fabrication phase. For instance, any malfunctioning component in a running vehicle at their actual use after fabrication, may threaten drivers, passengers and other people on road, and cause severe accidents affecting the infrastructures.

In an Industry 4.0 context, cyber-physical systems cover smart machines, storage systems and production facilities, data transmission networks and more – not just in one factory but across many. Together, the industrial internet escalates the threat of damage from cyber-attacks that may even block processing and workflow systems. Malicious attackers improve their tactics day by day, which may cause disruptions or longer outages at enormous cost. Industry 4.0 environments are prone to a huge number of security challenges as they are very complex systems. Hence, they need to be prepared to handle a wide variety of cybersecurity threats ^2^.

It is obvious that there is a continual pressure to ensure vehicles are correctly manufactured at the first time through; the costs, quantities and consumer exposure of warranty issues continue to increase; and the industry needs to work together to address these concerns. As a consequence of this trend, it has been reported that the testing, inspection and certification market in the automotive domain is expected to grow from USD 16.5 billion in 2020 to USD 20.4 billion by 2025, at a compound annual growth rate (CAGR) of 4.3% ^3^.

This paper aims to present the verification and validation (V&V) approach used for the purpose of quality control of automotive body-in-white, proposed as one of the thirteen use cases in the Verification and Validation of Automated Systems’ Safety and Security (VALU3S) project which is funded under the Horizon 2020-ECSEL-2019-RIA (Electronic Components and Systems for European Leadership 2019 call for Research and Innovation Action) programme. The use case illustrated in this paper aims to demonstrate and evaluate the automated robot inspection cell for quality control of automotive body-in-white. The paper introduces the VALU3S approach and its application within the context of the targeted use case, which is active in a real automotive industrial setting. The V&V methods, tools and concepts are presented with respect to the safety, security and privacy requirements of this industrial setting, which is active in Otokar’s premises in Sakarya, Turkey. Note that this paper mainly presents the novel approach addressed within the VALU3S project and the joint study of project partners which is composed of three research-oriented small or medium enterprizes (SMEs, Ergunler R&D Co., Inovasyon Co. and Techy Co.), a renowned unviersity (Eskisehir Osmangazi University) and a distinguished large industrial organization in automotive sector (Otokar). Furthermore, technical progress elaboration and comparison with alternative approaches are left to future studies. It is believed that this paper will shed light on any strategic and visionary discussions being held in the context of V&V to achieve more trustworthy industrial settings in the fields of Industry 4.0, smart factories and automotive fabrication.

The organization of this paper is as follows. Section 2 presents the overall V&V approach tackled in the VALU3S project. Section 4 elaborates the targeted use case and presents the overall cyber-physical system and the underlying solutions to achieve the safety, cybersecurity and privacy (SCP) requirements of a typical quality inspection system in automotive manufacturing. Section 3 revisits the current state-of-the-art and links the presented work with ECSEL priority areas. Finally, Section 5 concludes the paper with provisional objectives and future work.

## 2 A brief description and objectives of the VALU3S project

With recent developments in electronics in the last decade sensors and controllers have become more affordable. These technological developments in electronic components improve many automation systems for the benefit of both business and social life, in fields like automated driving systems, smart robotic systems in industry, smart transportation solutions and similar smart cyber-physical systems. Therewithal, data transfer rates have also increased enormously allowing real-time data collection from automated systems. Moreover, super-computers in a network or the cloud are becoming widespread to monitor and even control systems, observe the current situation of systems and optimize processes according to collected real-time data. Even connected automation systems perform well as prototypes, as they need to tackle security and safety requirements before their transformation into final products. The VALU3S project focuses on developing and improving techniques for the V&V of automated systems taking safety, cybersecurity, and privacy (SCP) requirements into account.

Manufacturers allocate an enormous amount of time and effort for the research into, and development of, automated systems. These systems are expected to pass verification and validation processes, including regulatory or standards compliance checks, before in-house use or commercial sale. However, it is not a trivial task to make sure that the developed systems work as intended and comply with regulations and standards specifications. The V&V process can be challenging, especially as system complexity increases and the system consists of more integrated and interconnected components.

Effective V&V methods and tools are extremely useful for automated system manufacturers. VALU3S aims to design, implement, and evaluate state-of-the-art V&V methods and tools to reduce the time and cost needed to verify and validate automated systems through SCP requirements. As planned, the implementation of V&V methods, improved process workflows, and tools will be demonstrated by 13 use cases with specific SCP requirements from the six domains of the automotive, industrial robotics, agriculture, aerospace, railway, and health sectors. In total, 42 participants, consisting of SMEs, universities, and research organizations, large enterprizes, and end-users, are working together under the VALU3S project to deliver a common V&V platform for cyber-physical systems.

All VALU3S participants aim to achieve eight objectives throughout the project: (i) developing a multi-layered framework enabling more effective verification and validation, (ii) overcoming the SCP gaps and limitations of cyber-physical systems, (iii) presenting a novel, standards-compliant V&V workflow that is generic to reference methods in selected cyber-physical domains, (iv) demonstrating, verifying and validating the usefulness and wider acceptance of the proposed framework by realistic pilots, (v) suggesting and validating new as well as state-of-the-art evaluation scenarios for safety, cybersecurity and privacy, (vi) developing and improving V&V tools and evaluation criteria, (vii) revisiting and identifying the weaknesses of relevant safety and security standards and developing a concrete strategy to influence the development of new standards, and (viii) presenting guidelines for end-users and practitioners to disseminate the project results for the purpose of increasing the awareness on the importance of conducting SCP-driven V&V.

To achieve the objectives, project partners work within seven work packages. The first work package includes the instantiation of use-cases and the creation of evaluation scenarios. The system requirements related to SCP and evaluation scenarios are derived for each use-case. Then, the test cases are designed by considering the related test requirements. The second work package is to design a multi-dimensional layered framework for the V&V of automated systems. In this task, a repository storing the elements of the framework is created, and all of the use-cases and test cases are mapped to the elements of the framework. The third work package is mainly focused on designing SCP V&V methods for automated systems in order to reduce the time and cost of V&V processes. The new methods are now being created as a combination of methods, a chain of methods, as well as by improving the current methods. The implementation of tailored V&V process workflows and tools are carried out in the fourth work package. In the fifth work package, project participants aim to demonstrate the implementation of the V&V process workflows to the use-cases and presenting the results for the evaluation of framework usability. The sixth and fifth work packages are, respectively, dissemination/exploration/standardization and project management which will continue throughout the entire project period.

The use-case, so called *Automated robot inspection cell for quality control of automotive body-in-white*, has a sufficiently rich and complex system architecture that allows the testing of V&V methods and tools for SCP in different aspects. During the project, V&V methods and tools are planned to be applied to the use-case. It is expected that the system will be improved to resolve deficiencies by the assistance of the VALU3S V&V framework. Note that the V&V processes can be re-employed, and adapted if needed, to evaluate the success of the improvements. Not only will the V&V methods, tools and the framework will be tested on the use-case, but these will also contribute to the development of the system reliability for SCP in a generalized manner for its exploitation in other domains of Industry 4.0.

### 2.1 Linking with ECSEL priority areas

The proposed work presented in this paper is built on the 2019 version of the Electronic Components and Systems (ECS) Strategic Roadmap Agenda (SRA) ^4^ which presented a common framework for research, development and innovation (RDI), identifying technology challenges and research priorities along 10 key application areas and essential capabilities. Among these, *Digital Industry* is set as one of the vertical key application areas which is highly relevant with the use case mentioned in Section 4. *Safety, Security and Reliability*, *Systems and Components: Architecture, Design and Integration* and *Connectivity and Interoperability* are the three most relevant horizontal capabilities as mentioned in 4.

**Figure 1.**
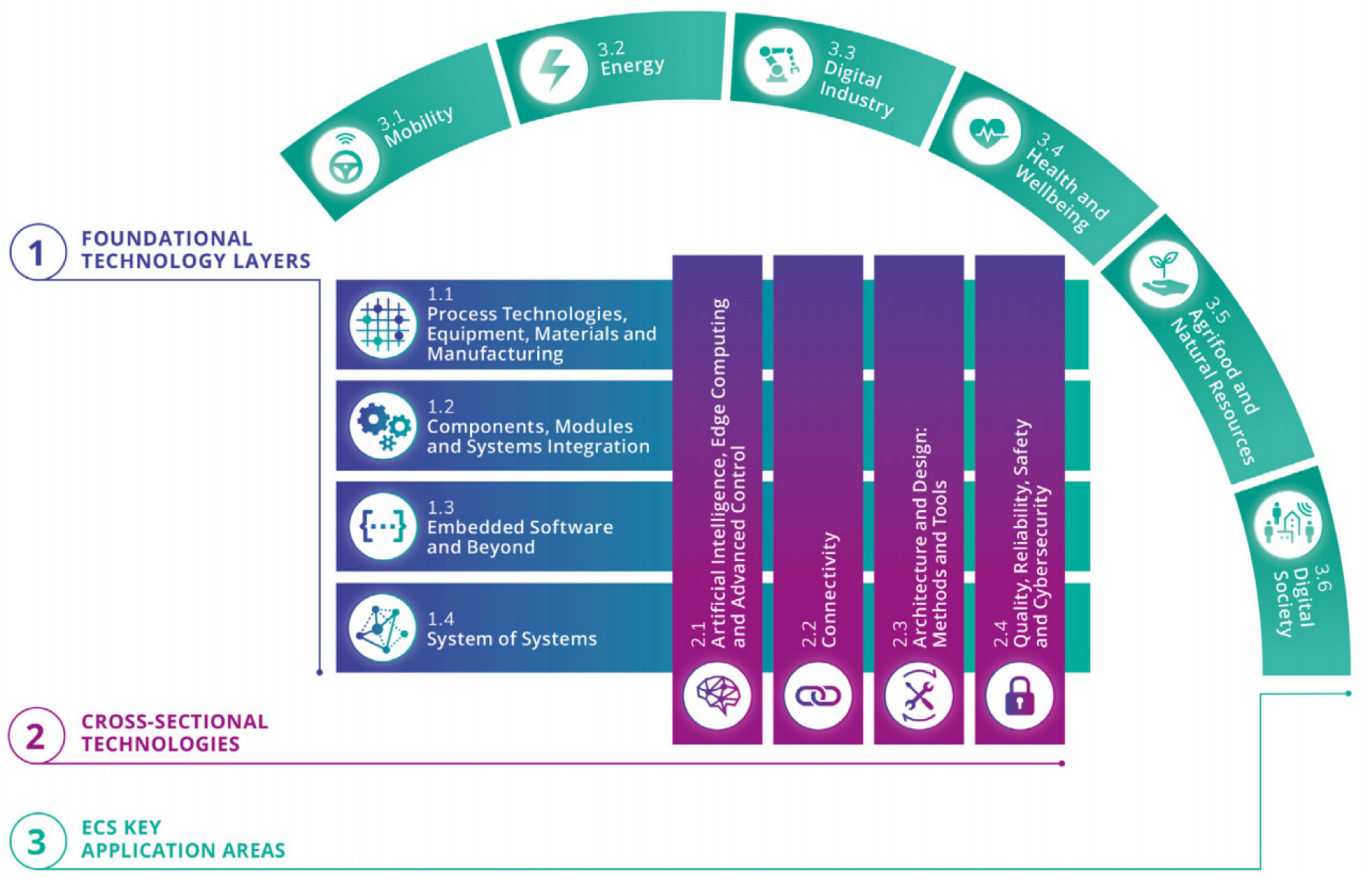
The new structure of the ECS-SRIA ^5^.

The three industry associations AENEAS ( **A**ssociation for **E**uropean **N**ano **E**lectronics **A**ctivitie **S**), ARTEMIS-IA ( **A**dvanced **R**esearch and **T**echnology for **EM**bedded **I**ntelligent **S**ystems - **I**ndustry **A**ssociation) and EPoSS ( **E**uropean Technology **P**latform **o**n **S**mart **S**ystems Integration) have joined forces to publish the new roadmap in 2021, namely the Electronic Components and Systems Strategic Research and Innovation Agenda (ECS-SRIA) ^5^. Similar to the previous ones, the new roadmap re-identifies the "bricks" of the ECS stack which are classified as I) devices; II) components; III) modules; IV) systems; and V) system of systems. As depicted in Figure 1, the digital industry is still one of the key application areas which reflects the main scope of this paper and covers the "foundational technology layers" and "cross-sectional technologies". Similar to the previous conceptual framework of ECS-SRIA, the presented work relies mainly on two horizontal foundational technology layers: "components, modules, and systems integration" and "embedded software and beyond". Vertically, the presented work is highly related to the cross-sectional technologies: "quality, reliability, safety and cybersecurity" and "architecture and design: methods and tools".

## 3 State-of-the-art

### 3.1 Simulation based fault injection tool for industrial robots

Fault injection is one of the main methods for testing autonomous systems. Since the late 90s, methodologies for different fault injection techniques have been presented. With the enhancement of complex systems, simulation-based fault injection has gained importance in system verification. In this area, new studies and tools are still being presented. In current state-of-the-art studies, fault injection description language (FIDL) is a compile-time fault injection tool that provides a description language for software fault injection. FIDL enables the creation of source code for new fault models ^6^. Furthermore, library-level fault injector (LFI) tool is presented for extracting errors in restricted libraries and LFI generates faults for libraries by analyzing faulty profiles of libraries and runs exhaustive injection ^7^.

The generic software fault injection technique (G-SWFIT) is another important technique that provides fault classification enabling the analysis of many code sources. G-SWFIT modifies ready-to-run binary code with specific changes and intends to generate faults in low level. This enables the emulation of the intended fault in high-level analyses ^8^. SAFE ( **S**oftw **A**re **F**ault **E**mulator) tool, another fault injection utility, has a similar concept with G-SWFIT. However, SAFE tool provides software-based fault injection by enabling the user to select a subset of faults in order to inject residual software faults ^9^. ProFIPy is a promising software for fault injection tool which has the ability to scan the workload and the source code for identifying fault injection points. ProFIPy is able to mutate Python codes and analyze the logs of faulty systems ^10^. Additionally, Gazebo Simulator provides functionalities for software testing. Gazebo delivers unit, integration and regression tests for specific robots ^11^. However, in some cases, software fault injection methods cannot provide sufficient mechanisms. For instance, algorithmic faults may not be found by software-based fault injection techniques. The verification of the system can be analyzed with the field data, and improved with simulation-based testing.

### 3.2 Artificial intelligence (AI)/machine learning (ML)

The non-deterministic nature of deep neural networks (DNN) yields to failures in generalizations. From a safety perspective ISO 26262 contradicts the black-box nature of DNNs ^12^. The three most important system concepts in V&V are safety, robustness and reliability. Safety is defined as "freedom from unacceptable risk of physical injury or of damage to the health of people" in the IEC 61508 standard ^13^. Robustness is defined as "the degree to which a component can function correctly in the presence of invalid inputs or stressful environmental conditions" ^14^. Reliability is delineated as "the probability that a component performs its required functions for a desired period of time without failure in specified environments with a desired confidence".

Aligned with the safety, robustness and reliability definitions, challenges for the V&V of ML-based systems can be I) the vast size of input spaces, II) invalid inputs or ragged environmental conditions, III) a combination of conventional components with ML-based ones, IV) the uncertain nature of ML systems, V) requirements and specification challenges, VI) designing test cases for such systems, and VII) threats coming from adversarial examples ^12^. Accidental risks are also present in AI-based systems, such as negative side effects, reward hacking, scalable oversight, safe explorations and robustness to distributional shifts ^15^. One major approach to mitigate some of these issues is building safety encapsulations for the system under testing, where unsafe and safe regions are defined and the system is triggered when a transition to an unsafe region occurs. This approach is named as the *safety cage* ^16^. In order to generate a large number of high-quality test cases based on formal requirement specifications and learning models, learning-based testing (LBT) may be employed ^17^. The LBT approach is an iterative method for automatic black-box testing and includes all of the major testing steps: test case generation, execution and evaluation ^18, 19^. It expands the specification-based testing framework by incorporating a learner and the corresponding system model.

The fault injection (FI) method, where explicit faults are introduced into a system-under-testing and the resulting behaviors are observed, can be categorized into two classes: execution-based and simulation-based methods ^20^. More recently, however, model-implemented (MIFI) and virtualization-based (VFI) techniques have also been introduced ^21^. In a cyber-physical system, faults yield errors and errors create failures within a chain effect. As a self-learning ML technique, reinforcement learning (RL) is proposed to overcome the chain effect where an agent explores its environment by observation and a reward function guiding the agent is defined. Lately, RL-based methods have been introduced as they explore fault spaces more efficiently and improve fault coverage ^22^. Other ML-based techniques for FI include Bayesian networks ^23^ and k-means clustering ^24^.

### 3.3 Safety trajectory optimization (formal verification)

Advanced robotics applications need to overcome safety issues for users, the environment, and the robot itself. In the literature there are several sources which address the safety issue for industrial robots: survey papers ^25– 27^, projects ^28^, research papers ^29– 32^, and standards (ISO 10218 ^33, 34^, ISO/TS 15066 ^35^). Although safety verification is very important, especially in robotic systems, it is a challenging task to verify and validate the safety of the robotic systems since autonomous robotic systems are complex, hybrid, and often safety-critical. Though commonly used, testing and simulation alone are insufficient to ensure correctness or to provide sufficient evidence for the certification of autonomous robotic systems. Therefore, formal methods for autonomous robotic systems have received some attention in the literature, but there is a need for more formal methods that can be used in these systems and for the better representation of these methods in real robotic systems ^36^.

Model checking is one of the most widely used approaches to verify robotics systems. It is widely used with various formalisms, including timed-automata, timed Petri Nets, etc. Some model checking tools handle timed models like UPPAAL ^37^ (a model checker tool jointly developed by researchers at **UPP**sala University and **AAL**borg University) or probabilistic models like PRISM ^38^ ( **PR**obab **I**listic **S**ymbolic **M**odel Checker). While timed models are feasible to represent the safe behavior of robotic systems, the probability is very convenient for modeling the environment of the robot system. There are case studies, including the model checking of robotic systems ^39^ proposes an approach to model and verify the software of a physical mobile robot using model checking ^40^. presents a case study on the formal verification of a high-level planner for an autonomous personal robotic assistant. A model of the robot and its environment are verified by using model checking.

Many studies argue that using the model checking approach in combination with other methods can make the verification more effective ^41^. provides a comparison between model checking and runtime verification and concludes that the two methods can be found in a combined manner, especially in highly dynamic systems such as an adaptive and self-organizing system like autonomous robotic systems. In 42, V&V of human-robot teams is implemented by the combination of model checking and simulation-based testing methods ^43^. presents a method as the combination of model checking and runtime verification for safe robotics.

### 3.4 Cyber-physical security

In both traditional and recent industrial Internet applications, encryption is the first line of defence to protect the confidentiality of the transmitted data. Encryption can handle attacks such as data injection, spoofing, and eavesdropping, but the low-power and low-cost requirements of internet of things (IoT) devices result in difficulties of finding reliable high-entropy sources for cryptographic operations (e.g. key generation, symmetric encryption). One possible solution is to utilize the hardware entropy, e.g. physical unclonable function (PUF), which depends on the unique hardware structure as source of randomness to generate the keys ^44^. Physical layer security is another solution since it provides a low-cost and reliable source to generate the secret key. This is because the channel information from the established connection between a sender and receiver pair, in a typical cyber-physical setting, is unique and can be leveraged as a source of high entropy.

Existing physical layer security solutions, either applied for single devices or any IoT framework, are still vulnerable to cloning and cryptanalysis attacks as they are providing mostly static solutions ^45^. One of the leading solutions is PUF-based integrated circuit (IC) components combined with fuzzy extractors aiming to increase resilience in interferencing errors and side channel attacks ^46^. However, these solutions do not give the guarantee of security-by-design as no one can be sure about the security leakages at production stages. Recent studies cope with this problem through a clever approach where hardware secure modules (HSMs) generate cryptographic one-time passwords, securing any point-to-point communication by benefiting from true random number generators ^47, 48^. Since such systems rely on fast and truly random and unpredictable number generation implemented at the field-programmable gate array (FPGA) level, the attack surface at hardware level is protected against any tampering, denial of service or phishing attacks because the entire duplex communication link is encapsulated by one-time and randomly created keys and very fast symmetric or asymmetric encryption.

The European Network and Information Security Agency (ENISA) presented a threat taxonomy which covers food practices for the security of the IoT in the context of smart manufacturing ^49^. The taxonomy presents the threat landscape in six layers, covering I) Level 0: Manufacturing processes and equipment (machines, robots); II) Level 1: Industrial IoT devices (IIoT) - sensors and actuators; III) Level 2: Industrial control devices and systems; IV) Level 3: Manufacturing operations systems and IIoT platforms; V) Level 4: Enterprise operations systems; VI) Level 5: Third party services. The countermeasures are categorized in eight classes including I) eavesdropping/interception/hijacking like man-in-the-middle attack or network reconnaissance; II) unintentional damages like accidents or human errors; III) outages like power cuts or communication network disabilities; IV) disasters like environmental or natural catastrophes; V) nefarious activities like manipulation of hardware and software or denial of service (DoS); VI) physical attacks like sabotage and vandalism; VII) failures/malfunctions like software and hardware vulnerabilities; and VIII) legal, for example violation of rules and breach of legislation or abuse of personal data. There exist holistic cyber-physical threat models in the automotive sector ^50, 51^ which address the cyber-physical vulnerabilities within the ENISA context. All these studies are very broad in context and there have been many holistic solutions. However, there still exists a great demand for integrated solutions, covering both hardware and software-based cyber-physical resilience, which are incorporated with effective verification and validation schemes.

## 4 Use case: automated robot inspection cell for the quality control of automotive body-in-white

**Figure 2.**
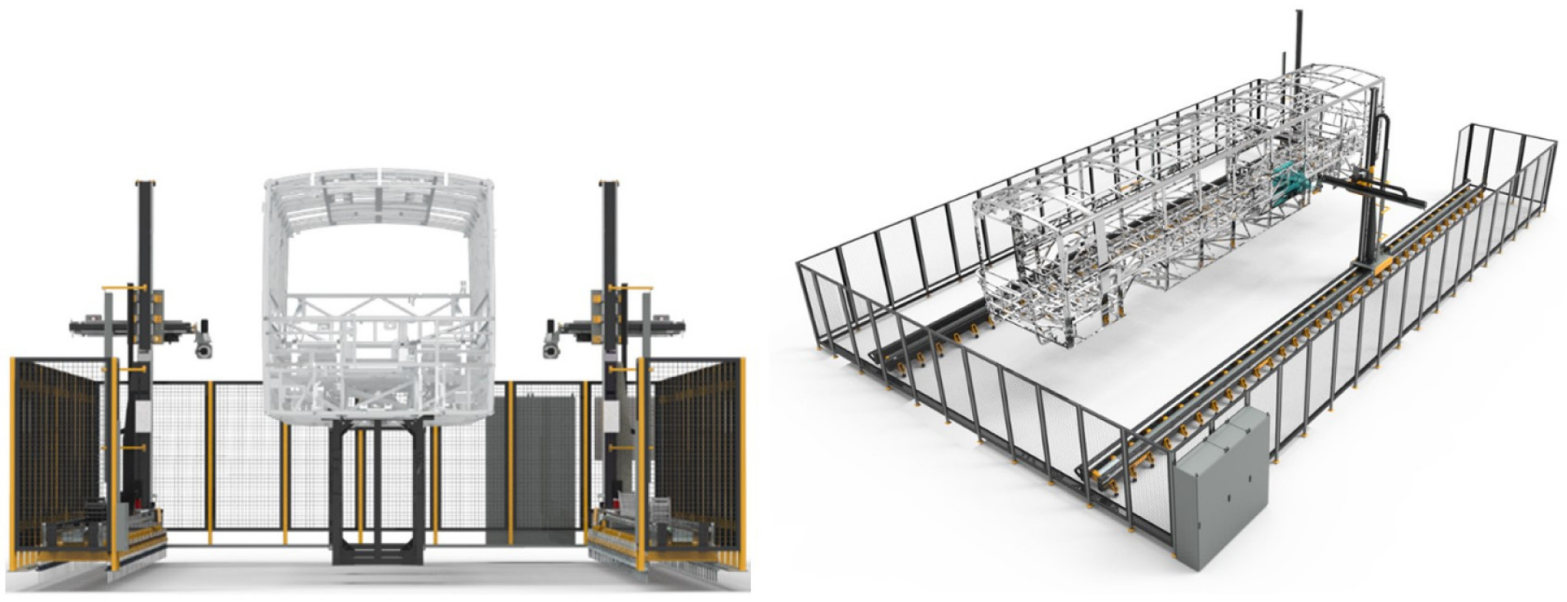
Robotic inspection cell for quality control.

The targeted use case focuses on a novel system using new visual inspection techniques to shorten the duration of existence control of the vehicles’ parts and components for better automotive body-in-white inspection (see Figure 2). The baseline of this use case is to provide a better fault-tolerant production system to achieve better quality control. Control of the existence of 2500–3000 body parts is planned to be executed fully automatically by a cartesian robot and camera sensor system. Using the computer-aided design (CAD) data for the vehicle, digital twin software developed by OTOKAR, which runs in a server, determines the safe robot trajectory points to check the existence of all vehicle parts. For each point stored in the server, the software positions the virtual camera in the CAD environment and renders a set of virtual two-dimensional (2D) images through a trajectory route. 2D images are also stored in the server. Processing the trajectory points as an input from the server, the software running in the system computer controls the axis motors via a programmable logic controller (PLC) and drivers to position the cartesian robots in its real production environment. For each positioned camera, sensors capture 2D images from the real production environment. A presence-absence check is performed by means of comparing the synthetic 2D image stored in the server and the actual image data obtained from the camera system. The quality reports and the system status data are finally stored for the quality control team which is responsible for giving the final confirmation.

### 4.1 Cyber-physical system overview and V&V needs

To ensure that VALU3S technology is applicable to the robot inspection cell for quality control, in this use case, we cover an automated fault and attack injection, specifically for controlling the entire industrial automation line. The existing quality check processes are still very time-consuming and ineffective and are not covering the safety requirements coherently. Additionally, quality checks in existing manufacturing environments are not very responsive and not adaptive to online sensing. The cyber-physical system works in S *top* & *Go* mode to minimize the physical threats. In this case study, there is a need to increase the autonomy of the system by ensuring the safety of the system. For this purpose, autonomous trajectory generation methods, optimized according to time and safety, are planned to be developed in this use case. In addition, since the robotic system is able to perceive the current state of the environment in real-time a dynamic motion plan is implemented by considering the physical appearance of the operator in the environment. The safety of the system aims to be verified both in the current system and the system developed in the laboratory. The safety requirements cover the safety of the operator, robot and its apparatus as well as static objects in the workspace. In manual mode human safety is planned to be improved by maintaining secure communication with operator and robotic system to prevent any cyber attack.

**Figure 3.**
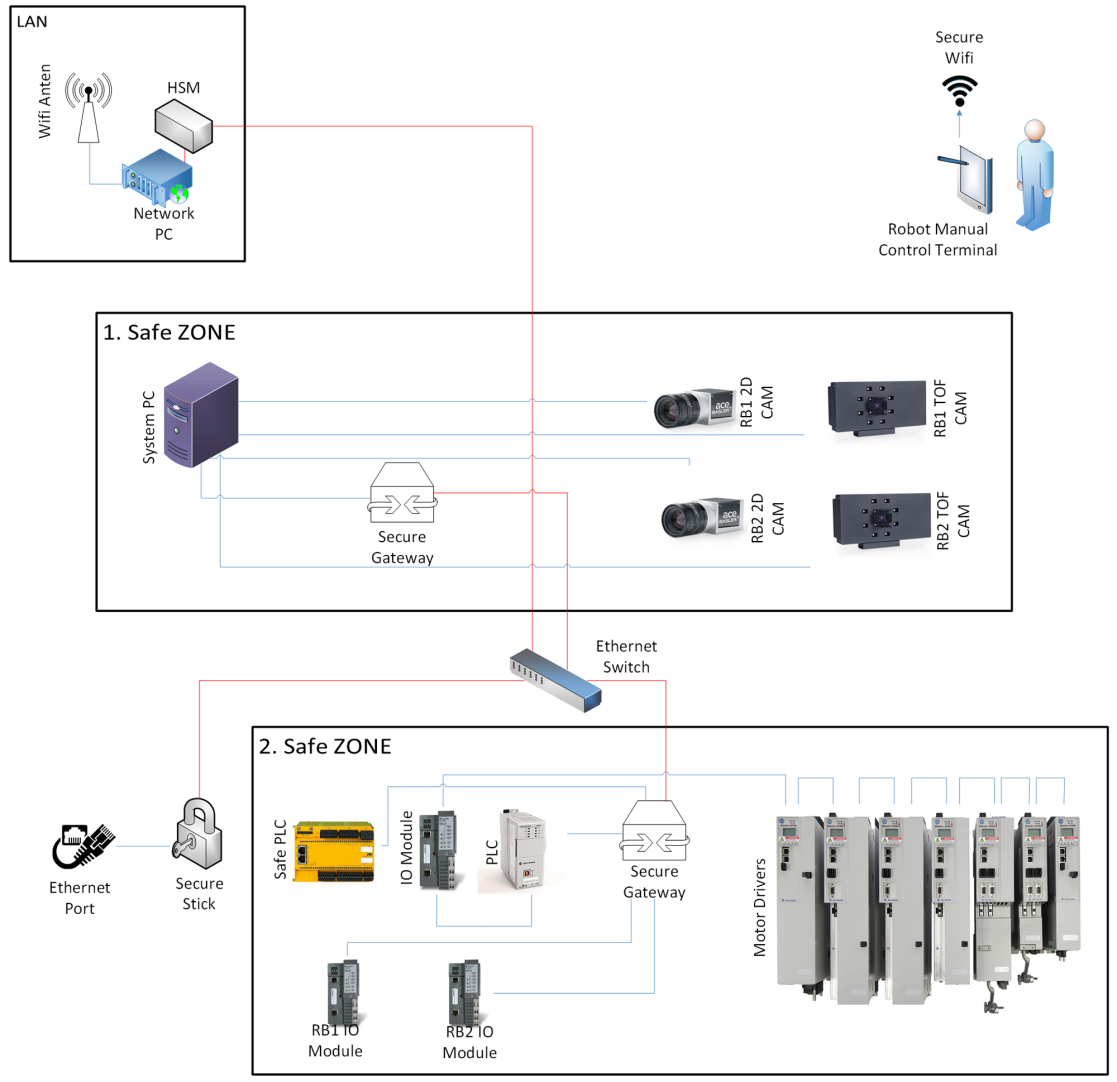
Detailed topology of the system showing data transfer protocols.

As presented in Figure 3, the topology of the target system is as follows: the system consists of two safe zones transmitting real time data to and receiving input data from a network PC located away from the system. There is a system PC, two time-of-flight (ToF) cameras and two 2D cameras in the first safe zone. The cameras directly transfer image data to the system PC. The system PC sends camera data to be stored via the first secure gateway, enabling encrypted data transmission to the network server PC. There is automation equipment in the second safe zone which consists of a PLC, I/O modules, safety PLC and motor drivers. I/O modules and motor drivers are directly connected to the PLC. Data obtained by the PLC is transmitted to the system PC through the second secure gateway in a an encrypted way. The transmitted data is decrypted in the first secure gateway and delivered to the system PC as decrypted. If the system data received from the PLC is required to be processed or stored in the network PC, then the system PC transmits the data through the first secure gateway and the ethernet switch as encrypted to the network PC. The received data is decrypted by the HSM which is installed on the network PC. The HSM is also used to produce tokens to encrypt or decrypt data that is sent to secure gateways. Through these encryption processes manipulation of system data and any privacy leakage can be prevented. The system is accessible through Wi-Fi or regular ethernet ports. Anomalies in network data transfer can be observed in case of a cyber attack, e.g. man-in-the-middle (MiTM) attack and address resolution protocol (ARP) poisoning.

The V&V of this system is vital because the targeted automated robot inspection cell is planned to be used online for mission-critical operations of manufacturing where many stakeholders, for instance third party collaborating organizations, should work together over a heterogeneous and secure platform. The common V&V framework proposed in VALU3S enables secure access for stakeholders, e.g. quality experts, to the proposed quality inspection system in order to monitor the results of the quality inspection analyzes in a trusted way.

### 4.2 Holistic security and V&V for a trusted cyber-physical system

Cyber-physical security is indispensable in any smart system where the generated and flowing data should be encrypted. This is a very natural need of any cyber-physical system where cryptographic operations, e.g. encryption/decryption, hashing, key generation, key management, and user authentication, take place. One of the basic principles of cryptography is that, according to Kerckhoff’s hypothesis ^52^, it is assumed that the overall security of any cryptosystem is completely dependent on the security of the key, and that all other parameters of the cryptosystem (including the cryptographic algorithm) are publicly observable. Thus, the cryptographic algorithms are assumed to be open as long as the key generation scheme is not secure. In real life, many systems actually use well-known symmetric and asymmetric cryptographic algorithms (advanced encryption standard [AES], triple data encryption standard [3DES], Rivest–Shamir–Adleman [RSA], elliptic curve data encryption standard [ECDSA], secure hash algorithm [SHA]) which have been applied in many dimensions, and all experts are aware of their strengths and weaknesses. It is mandatory that the presented cyber-physical systems in VALU3S should be resilient against cyber-physical threats and vulnerabilities. In this regard, security countermeasures are seriously introduced within the VALU3S context which can be categorized in two ways: I) holistic cyber-physical security and II) vulnerability assessment for the verification and validation of the cryptographic schemes.

**Figure 4.**
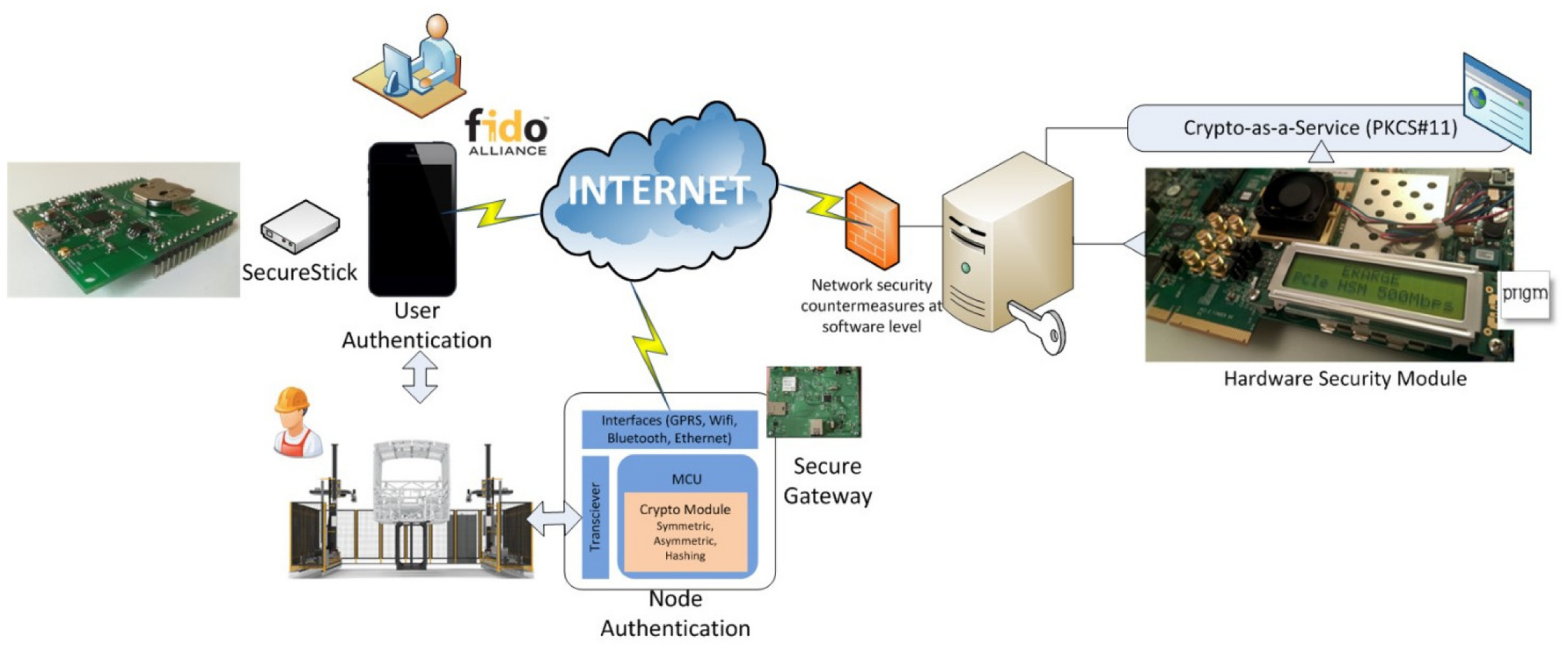
Hardware-based cyber-physical security.

***4.2.1 Holistic cyber-physical security.*** In VALU3S, cyber-physical security is tackled by a holistic approach which operates in a typical client-server architecture. As depicted in Figure 4, a HSM (branded as PRIGM©) is deployed at the server side connected to a server (e.g. identity management server), via its PCIex interface. The developed HSM is an advanced cryptographic device which is based on a strong theoretical background including true random number generation and vulnerability analysis. As patented ^53^, the HSM applies very advanced techniques to generate random numbers in a very fast and accurate way so that it brings practical advantages in the targeted smart system where where security and privacy preservation can be handled at system nodes.

On top of the physical layer of the HSM, crypto-as-a-service (CryptaaS) is positioned, and it is also connected virtually with the server. CryptaaS is a high-level software service which enables the basic functionalities of the HSM. CryptaaS provides software-level integration of cryptographic functions, fully compliant with the PKCS#11 standard ^54^. CryptaaS behaves as a supplementary tool to enable encryption before injecting any data to the cyber-physical system ( Section 4.1). The proposed scheme provides PKCS#11 standard (Cryptoki API) for its implementation via a LINUX driver written on both FPGA and Linux Ecosystem to execute basic cryptographic algorithms.

At client side, authentication is tackled in two dimensions. The first dimension is for person authentication which is based on a FIDO-compliant (Fast IDentity Online) authentication scheme. FIDO ^55^ authentication is known as a set of standards for fast, simple, and strong authentication which has been developed by the FIDO Alliance, an industry association with representatives from a wide range of organizations including Google, Microsoft, Mozilla, and Yubico. The FIDO protocol authenticates a user to a server, using a token (a USB token, namely SecureStick in VALU3S), in such a way that the user is not impersonated without being in possession of her/his token, even if the username and the password of that user have been compromised. The protocol runs between a user, a token, a FIDO Client embedded in the user’s web browser (e.g. main dashboard web interface), and a server (on which HSM and CryptaaS are orchestrated), after the establishment of a secure transport layer security (TLS) protocol between the last two entities.

The second dimension is for node authentication which is used for end-to-end security between sensors, actuators and any system components. As depicted in Figure 4, Secure Gateway receives the data from the cyber-physical infrastructure by RS485 protocol and the transceiver converts this data to a universal asynchronous receiver-transmitter (UART) component on the gateway. The received data is then sent to the micro-controller unit (MCU) in which a crypto-module is implemented. The crypto-module has symmetric and asymmetric cryptographic components as well as a hashing submodule. This module is the core for securing the transmitted data. Once secured, the data is published to the cloud via an IoT Broker (e.g.MQTT) channel. Here, general packet radio service (GPRS), Bluetooth and WiFi are the wireless communication interfaces and the Ethernet is used for wired communication. Secure Gateway enables the duplex secure communication providing bilateral data transmission between any node pairs.

***4.2.2 Vulnerability assessment for the verification and validation of the cryptographic key generation.*** Vulnerability analysis is complementary to cryptography. Holistic security countermeasures become stronger due to the vulnerability analysis that reveals the weaknesses of the existing cryptographic systems. The robustness of a crypto system depends on the key used, or in other words, the attacker’s ability to predict the key. The hardware-based vulnerability analysis proposed in VALU3S relies on four aspects:

- **True randomness:** There are two basic types of generators used to produce random sequences: true random number generators (TRNGs) and pseudorandom number generators (PRNGs). TRNGs generate random numbers from a physical process (thermal noise, the photoelectric effect, ring oscillator) rather than software-based entropy sources. While TRNGs take advantage of non-deterministic entropy sources, PRNGs generate bits in a deterministic manner. The PRNG-generated sequence is not truly random, because it is completely determined by an initial value, called the PRNG’s seed (which may include truly random values). PRNGs tend to benefit from the external source of randomness (e.g., mouse movements, delay between keyboard presses etc.) which are practical in use but predictable. In other words, an attacker can easily guess the outputs of a PRNG by applying statistical or machine learning methods so that the cryptographic keys or secrets (which uses the bit streams as outputs of PRNGs) also become guessable (the previous or next bits can be predicted). The first thing to apply for the vulnerability analysis of a cryptosystem is to check whether the system relies on a hardware-based random number generator (RNG) or not. Then, this RNG should be a TRNG. To meet the true randomness criteria, three test suites are applied on a sufficient length of bit sequences: I) NIST-800-22 Randomness Test Suite ^56^; II) DieHard Test Suite ^57^; III) Big Crush Test Suite ^58^.
- **Unpredictability:** The randomness test suites are useful but the main message given by these tests is that the tested sequence is NOT random. As they rely on statistical techniques the randomness test suites do not give any guarantee about the true randomness of a bit sequence. Even if a bit sequence fulfils all the randomness test suites, they cannot present any quantification about their predictability. In order to say that a TRNG is reliable, the preceding and following random bits generated by the TRNG cannot be predicted by any technique. Although alternative methods exist, the proposed predictability analysis was presented in 59. An ideal entropy source which is used as the core of a TRNG based on thresholding random noise should have constant power spectral density over its operating bandwidth. The proposed vulnerability analysis technique in VALU3S relies on the generation of independent and uniformly distributed bits, per-sample joint entropy of the generated bit sequence and the autocorrelation function of the output. Here, it is assumed that the underlying noise waveform is based on a continuous-time wide sense stationary Gaussian process, of which power spectral density is flat between two known frequencies. Given a continuous time wide-sense stationary flat band-limited Gaussian noise source, the test aims to investigate the necessary and sufficient conditions for generating independent and identically distributed random bits with uniform marginal probabilities via uniform sampling from this process and providing analytical and numerical results for the per-sample joint entropy. This is realized by preparing an equivalent setup in place of the original one in order to make the derivations simpler.
- **Irreproducibility & robustness:** The strength of a cryptosystem almost depends on the strength of the key used, or in other words on the difficulty for an attacker not only to predict the key but also assure the irreproducibility of the same bit sequence and recommend a robust design against attacks. The TRNGs should not rely on the deterministic sources, otherwise one can even predict the secret parameters of RNGs ^60^. In recent years, the majority of TRNGs rely on non-linear systems, e.g. chaotic RNGs. So, the vulnerability analysis of the TRNG is tackled so that the irreproducibility and robustness against attacks is jointly encountered. This is realized by a novel predicting system that is proposed to identify the security weaknesses of a chaotic RNG. Convergence of the predicting system is proved using auto-synchronization. A secret parameter of the target chaotic RNG is revealed where the public information is the design of the chaotic RNG and a scalar time series observed from the target chaotic system. Simulation and numerical results verifying the feasibility of the predicting system are also presented similarly in recent papers ^61– 63^ such that the next bit can be predicted as well while the same output bit sequence of the chaotic RNG can be regenerated.

### 4.3 Simulation-based fault injection tool (IMFIT) for industrial robots

**Figure 5.**
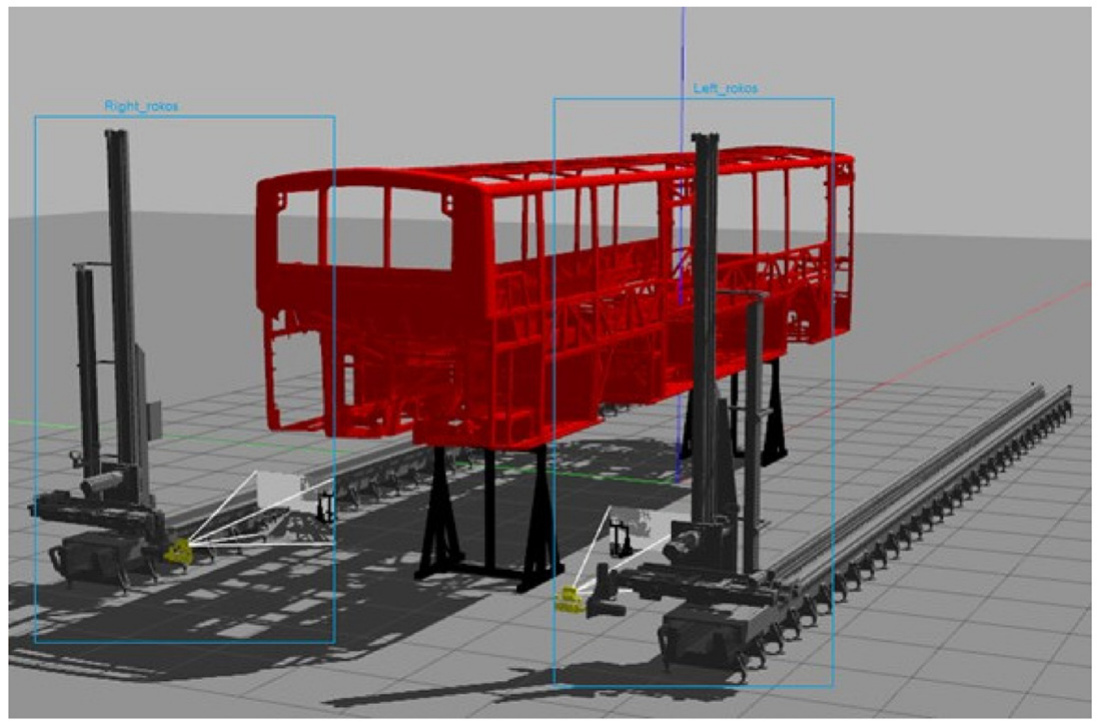
Gazebo virtual simulation world in the scope of the targeted use-case.

An IMFIT is tailored within the scope of the targeted use case as presented in Section 4.1. In this study, the real-world environment of the use case is transferred to the GAZEBOSim virtual environment as depicted in Figure 5 in a way to conduct tests and evaluation scenarios.

**Figure 6.**
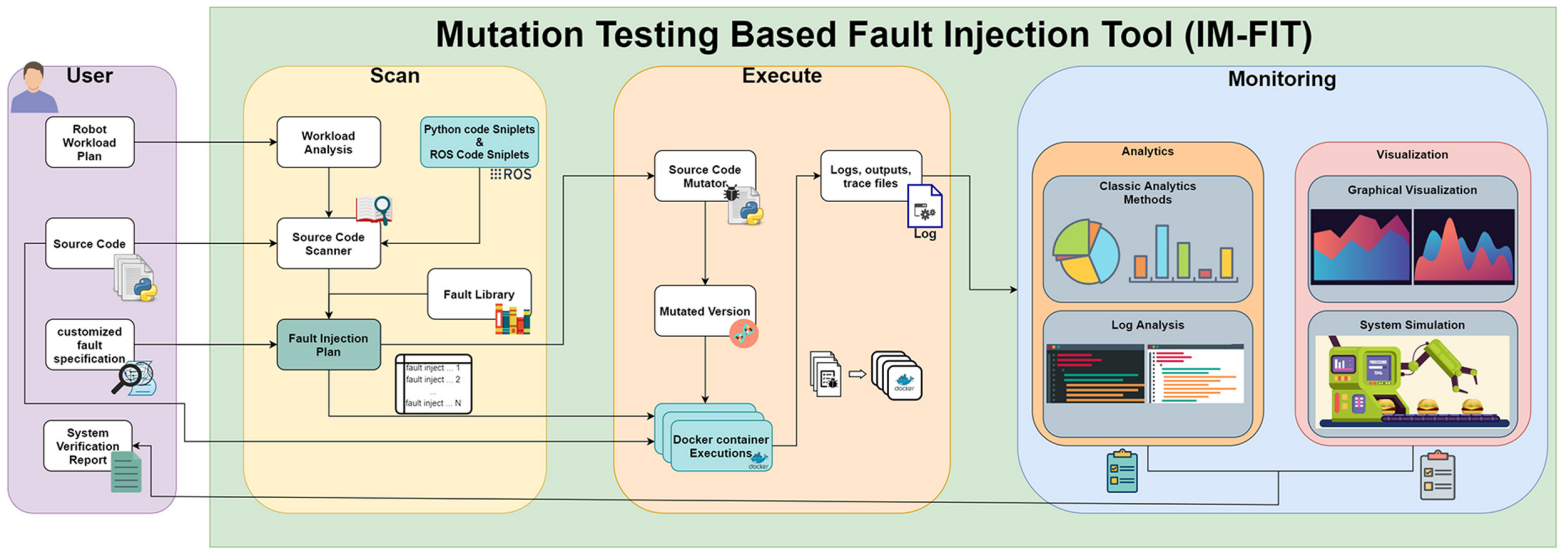
Architecture of the simulation-based fault injection tool for industrial robots.

An IMFIT is developed for the verification and validation of the targeted industrial robot systems. This system has elements that are specialized for testing industrial robots. The proposed fault injection mechanism is specialized for the fault tolerance test of the robotic system. As depicted in Figure 6, the IMFIT has three main modules. The first module is the scanning module in which the robot workload is analyzed. Libraries and functions that are used by the cyber-physical system are detected according to the workload on robotic arms. Specified parts in the source code are presented to the user as potential fault injection points. The user can select the type of the fault to be injected and monitored. Additionally, the user can define customized faults to inject into the source code. According to the selected fault injection plan, an execution plan is created. This plan is transformed into a docker plan. The second module is for the execution where the source code is mutated according to the fault injection plan. Mutated versions of the source code are stored separately for execution. According to the docker (container) plan, mutated codes and the original code are executed. Container executions are performed according to system specifications. After execution of the container, logs, outputs, bags and trace files are extracted for each scenario. At the third module, the so-called monitoring module, all these logs, outputs, bags and trace files are analyzed with classical analysis and log analysis methods. The robot parameter and actions are shown in graphics for detailed analysis. Moreover, faulty and original operations of the robot system are demonstrated with simulations. The monitoring module presents the results of the detailed analyses, metrics calculations, final reporting and the visualization of the safety assessments.

**Figure 7.**
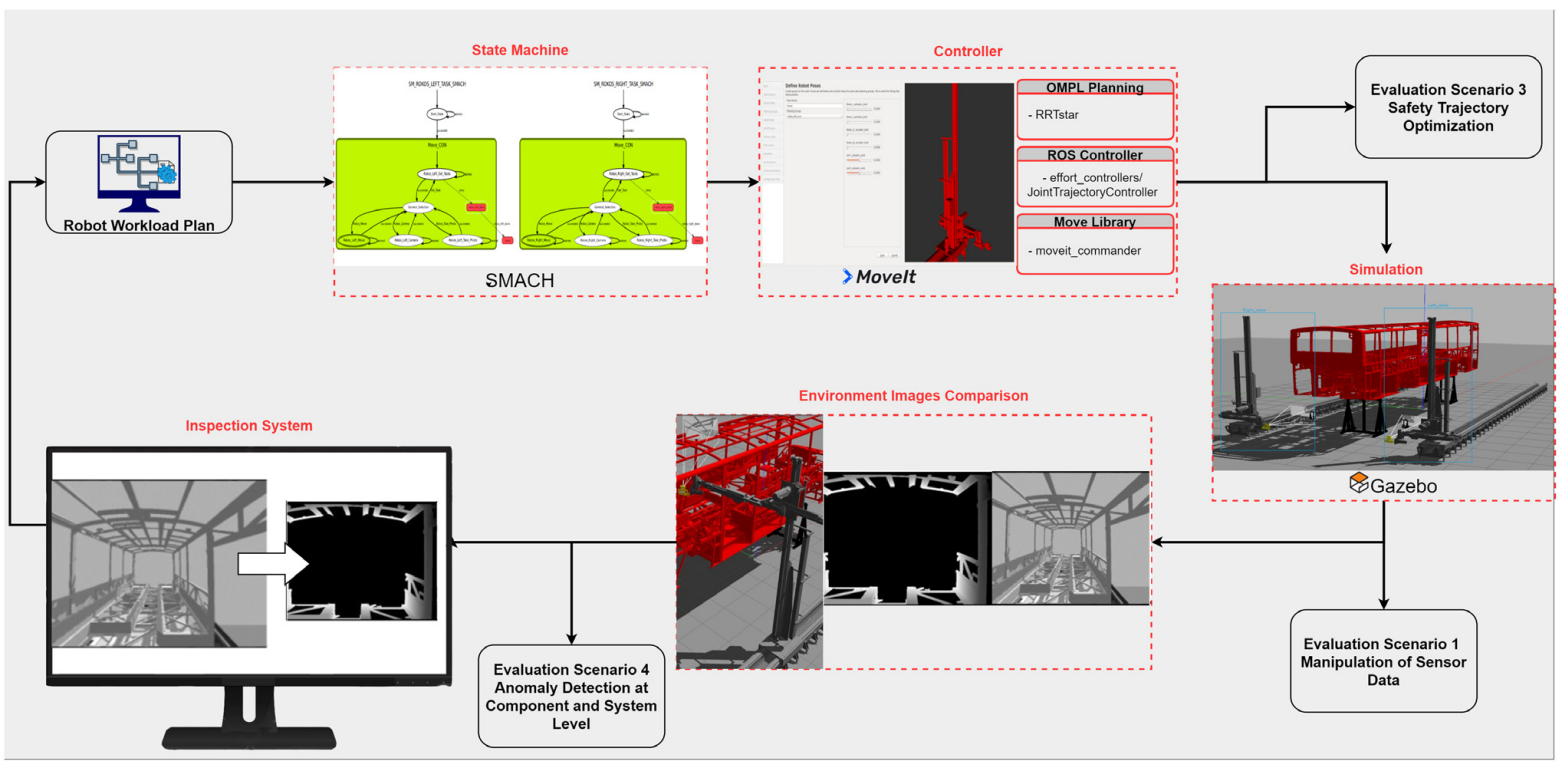
Architecture of the simulation environment for industrial robots.

The IMFIT is effectively applied to the targeted use case by simulating the automated robot inspection cell for quality control of automotive body-in-white. As illustrated in Figure 7, the architecture of the simulation environment is implemented by composing three tools: I) quality of the bus body-in-white is inspected with the finite state machine tool SMACH ^64^ that is used for workload implementation; II) MoveIT ^65^ is used for cartesian robots trajectory motion control and planning; and finally, III) GAZEBOsim ^66^ is used for the simulation of real environment. The IMFIT analyzes the workload and identifies the fault injection points in this scenario. For example, in GAZEBO simulations, the results of faulty behavior are observed through sensor data outputs. Images captured from the test environment show clear analysis results about system behavior anomalies. Detected anomalies at both component and system level are reported for further analysis.

**Figure 8.**
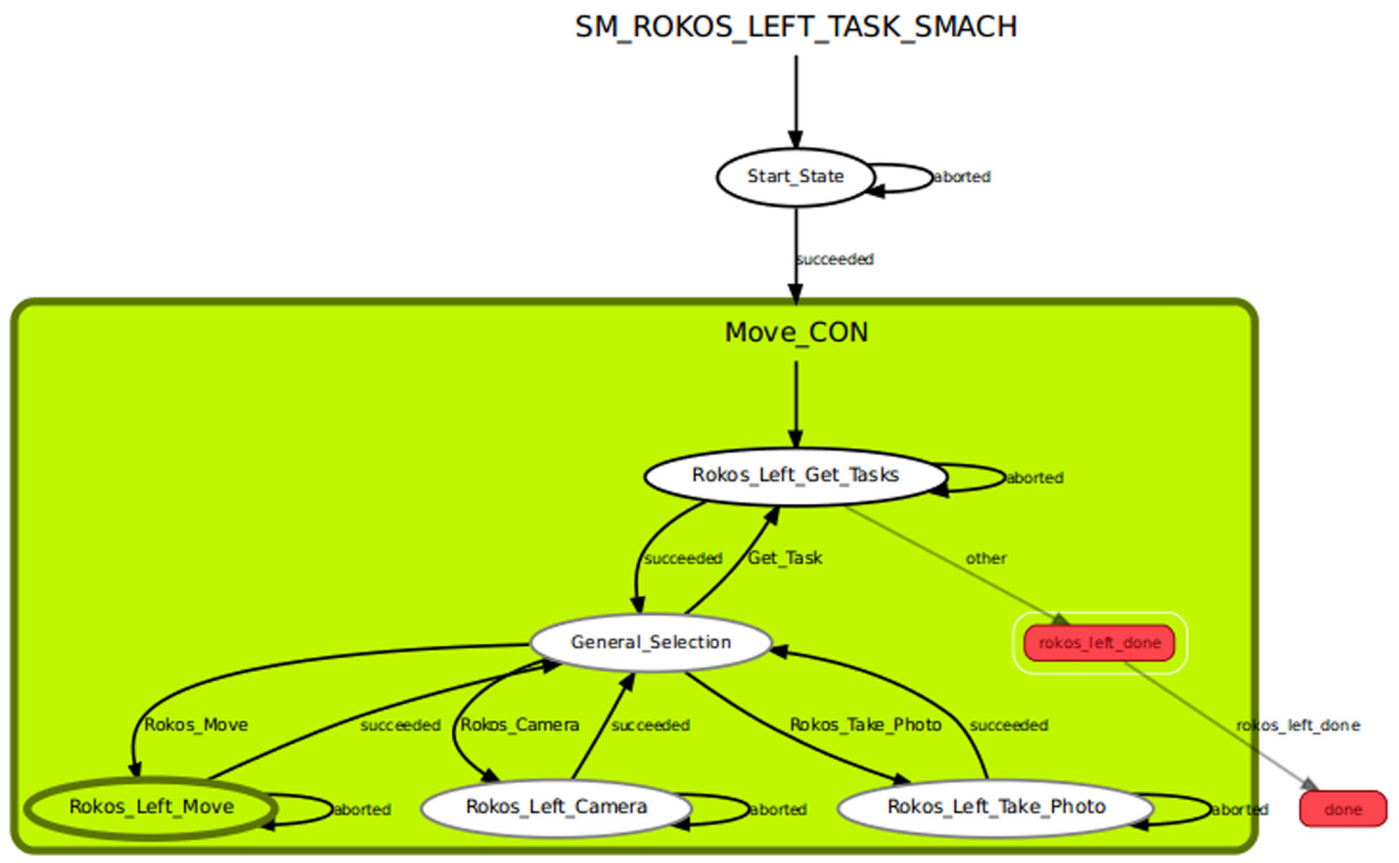
State machines in SMACH.

The scenario is created by using SMACH state machines ( Figure 8). SMACH is a library which is used to create hierarchical state machines. SMACH is an architecture that allows complex robot behaviors to be created simply at the task level. States are defined for the scenarios. The *receiving* state gets tasks by requesting tasks for the robotic arms of the cyber-physical system to the task service node. In the *motion* state, the created Cartesian path calculation and the execution function is called so that it can move dynamically to the positions as programmed in the tasks. The Cartesian path plans are logged for checking whether the stats are working properly. In the camera *move* state, orientation movements are given to the camera and the image *saver* node is created and called here. It saves the data read from the camera topics as an image file.

**Figure 9.**
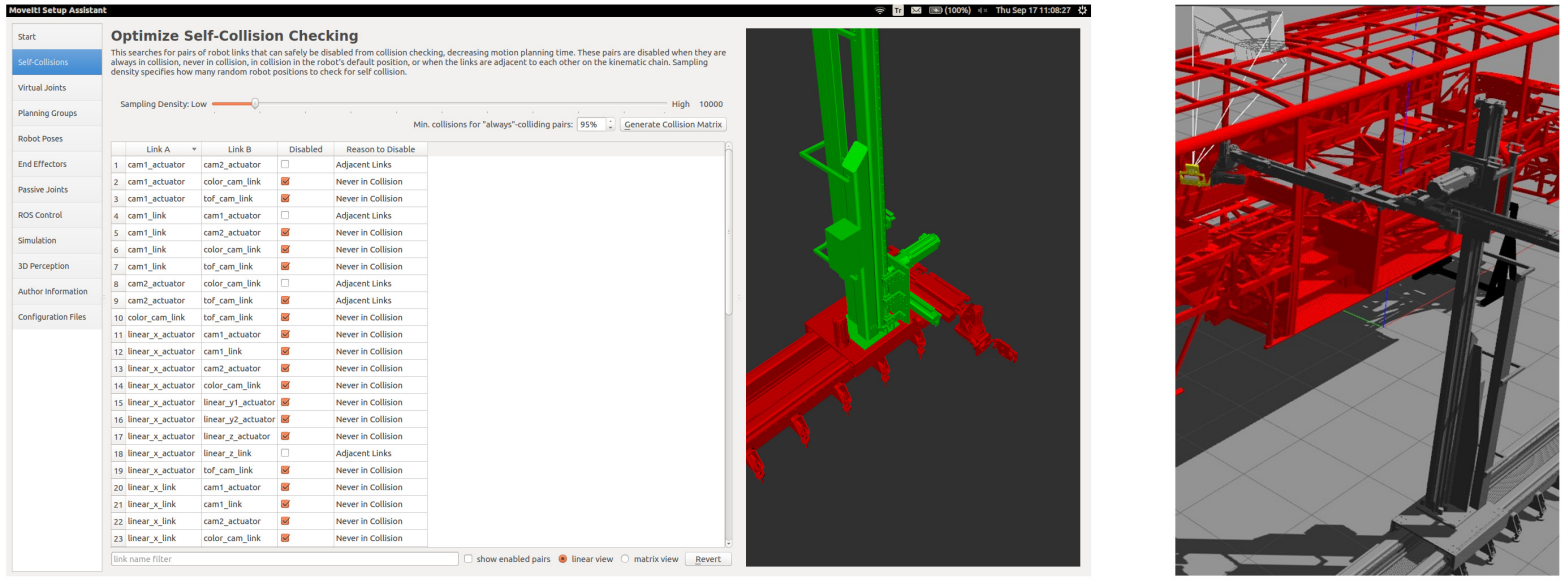
( **a**) MoveIt Setup Assistant ( **b**) robotic quality control system at goal position.

Moveit ^67^ is an easy-to-use open source robotic manipulation platform for designing prototypes and developing benchmarking algorithms. Gazebo, Robot Operating System (ROS) Control and MoveIt are used together to create a robust development platform. MoveIt Setup Assistant ( Figure 9 (a)) is a graphical user interface created to configure any robot for use with MoveIt. To configure it, the robot must have a semantic robot description format (SRDF) file. Collisions, virtual joints, planning groups, robot poses and ROS controllers can be configured via Moveit Setup Assistant. In this interface, an open motion planning library (OMPL) planner and relevant controllers are selected (rapidly-exploring random tree, [RRT] algorithm ^68^ for this scenario. See Section 4.4 for more details).

After the Moveit config files are created, Gazebo and MoveIt integration is performed. The *moveit_commander* library in MoveIt is used to move the configured robot to the Gazebo. Cartesian path calculation and the execution functions have been created so that the robot can dynamically navigate to the specified x, y, z coordinates. To provide Cartesian robot movements in Gazebo simulation, the *effort_controllers-joint_trajectory-controller* controller type is set. Appropriate proportional integral derivative (PID) parameters are set according to the minimum error between the goal position and the real position. After appropriate PID and inertia parameters are defined, robots move smoothly to the goal position. The movement of the robot in the goal position is shown in the Figure 9 (b).

**Figure 10.**
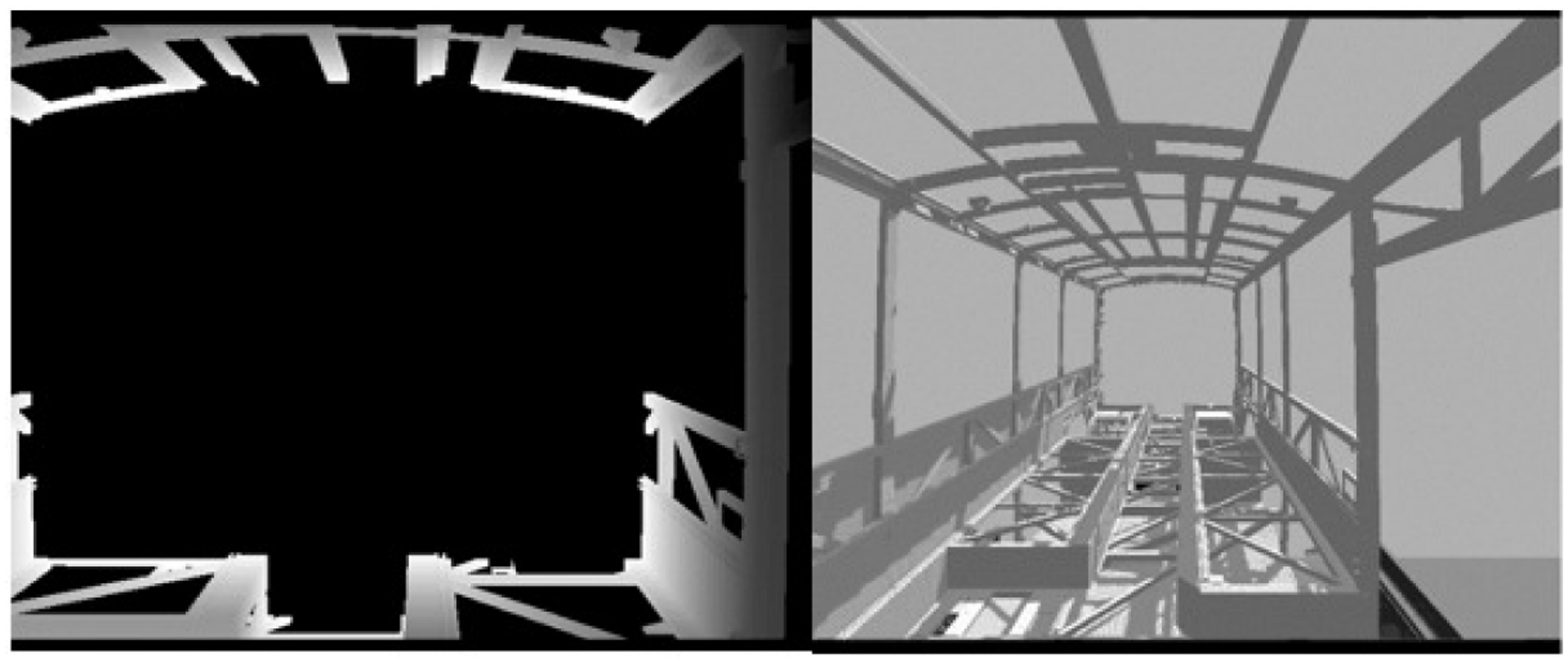
( **a**) Time of Flight (TOF) camera capture ( **b**) Area scan camera capture.

Gazebo is used as a simulation program, with physics engines allowing the user to mimic robots used in different surface environments. In the Gazebo environment, real-world models and coefficients, such as inter-material friction models, fluid dynamics, collision models, gravity coefficient, wind speed and direction, can be defined. Thus, the Gazebo simulation environment provides more realistic values for the sensors used in the robot. Typical uses for Gazebo are testing robotic algorithms, designing robots, and regression testing with realistic scenarios. For example, in the targeted use, two cameras are used in the simulated environment. One of them is the time of flight camera and the other is the area scan camera ( Figure 10 (a) and (b), respectively). Parameters of these camera sensors are defined based on real product cameras. This camera information can be used for anomaly detection at component and system level and for a case where the sensor data is intentionally manipulated.

As a result of this study, V&V of automated systems can be conducted by using IMFIT and the developed simulation environment. IMFIT is capable of facilitating and reducing cost, time and effort needed for the V&V activities for automated systems. Furthermore, IMFIT can be used in other domains like aerospace, automotive, medical etc. in VALU3S, and is compatible with other similar simulation environments like CoppeliaSim (formerly V-REP) ^69^.

### 4.4 Safety trajectory optimization

Trajectory planning enables a robot to move between two different configurations over time while fulfilling the constraints of the robot and its environment. The main difference between trajectory planning and path planning is time dependency. Path planning generates a geometric path from an initial to a final point through the waypoints while trajectory planning assigns a time law to the geometric path. In other words, several discrete segments of a path can be assumed as trajectories linked to form the path of motion. Trajectories are usually optimized by minimum time, energy, and/or jerk. Besides, trajectories are expected to be smooth so that this does not cause excessive accelerations of the actuators and vibrations of the mechanical structure.

**Figure 11.**
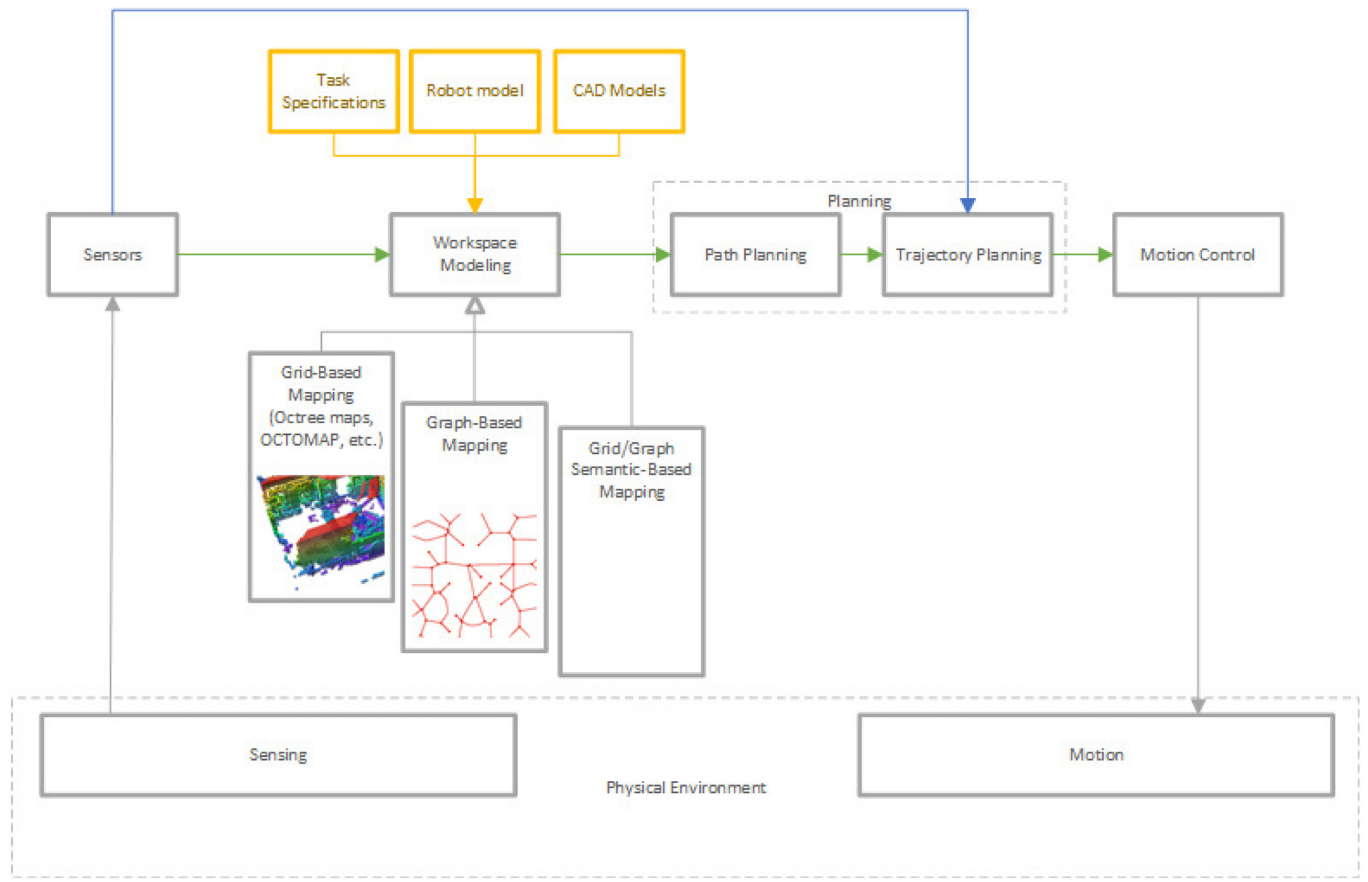
A control scheme for industrial robotic systems.

A control scheme including environment modeling and planning phases for an industrial robotic system is shown in Figure 11. In traditional applications, the trajectories are created by the manual teaching technique. The operator moves the robot from point to point, saving each position by using a teach pendant. Then, the robot can playback the motions. Another traditional application is simulation/offline programming of the robot. Once the CAD models of the environment and the robot are loaded into generic or manufacturer-supplied simulation software, the trajectories are created by using a virtual mockup of the robot and the task. However, in recent years, optimized trajectories are automatically provided for the given goal positions by the simulation software. In order to generate automatically provided trajectories, workspace modeling is required. Grid-based and graph-based methods are extensively used for workspace modeling. 3D environment modeling is generally represented by octrees that subdivide the represented space into eight octants. In current literature, there are efficient 3D mapping frameworks based on octrees ^70^.

There are various software libraries for planning, such as the Open Motion Planning Library (OMPL) ^71^, Stochastic Trajectory Optimization for Motion Planning (STOMP) ^72^, Search-Based Planning Library (SBPL) ^73^, and Covariant Hamiltonian Optimization for Motion Planning (CHOMP) ^74^. As addressed in Section 4.3 too, OMPL is an opensource motion planning library that primarily implements randomized motion planners. It includes many sample-based planning algorithms. One of the most well-known algorithms is Rapidly-exploring Random Trees (RRT) ^68^ which is easy to understand and implement. There are many variants of RRT. The other well-known planning algorithm is the Probabilistic Roadmap Method (PRM) ^75^ which also has many variants. STOMP is an optimization-based motion planner that can plan smooth trajectories for a robot arm, avoiding obstacles and optimizing robot constraints. SBPL provides a generic set of motion planners using search-based planning, and CHOMP is a novel gradient-based trajectory optimization method.

Moreover, trajectory planning directly affects the system safety which is a key factor in V&V of the targeted cyberphysical system. The planning algorithm has to generate path/trajectory plans which enable the robot to avoid damage to humans, other equipment in the workspace, or the robot itself. Industrial robot systems are expected to comply with strict safety requirements defined in the robot standards ISO 10218-1 ^33^ and ISO 10218-2 ^34^, and the most recent technical specification ISO/TS 15066 ^35^. The V&V methods can be applied at different stages of the robot software, including trajectory planning and trajectory tracking motion under the uncertainties of sensors, drivers, and the physical environment. Simulation-based verification is widely preferred in robotic systems. However, many formal V&V methods are employed for robotic systems including model checking, theorem proving, run-time verification monitoring, and the combination of different formalisms ^32^. Throughout the implementation of formal verification of robotic systems, many generic model checker tools can be used like UPPAAL and PRISM.

**Figure 12.**
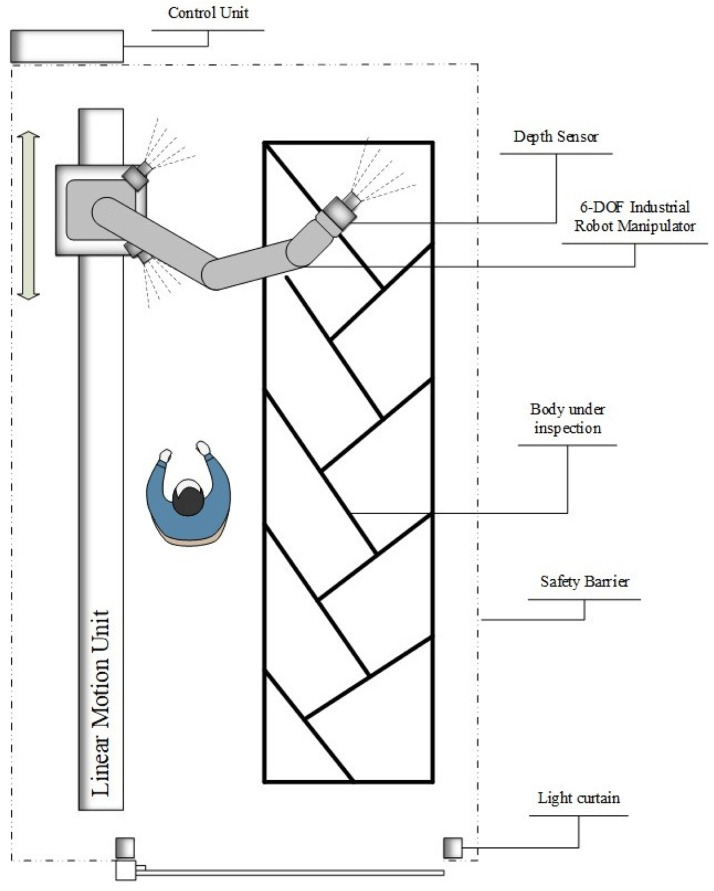
Experimental system as a small-scale mock-up of robotic inspection cell for quality control.

The robotic inspection cell for quality control has already been actively used in the production process. The design of safety-optimized trajectory planners and verification tools requires a structurally-controllable and easily-configurable test environment (e.g. at Technology Readiness Level 5 or higher). Therefore, an experimental system as a smallscale mockup of robotic inspection cell for quality control is planned to be constructed as illustrated in Figure 12. The experimental system can be used for developing and pre-testing effective safety-optimized trajectory planners and V&V methods.

### 4.5 AI-enabled techniques in VI&V

**Figure 13.**
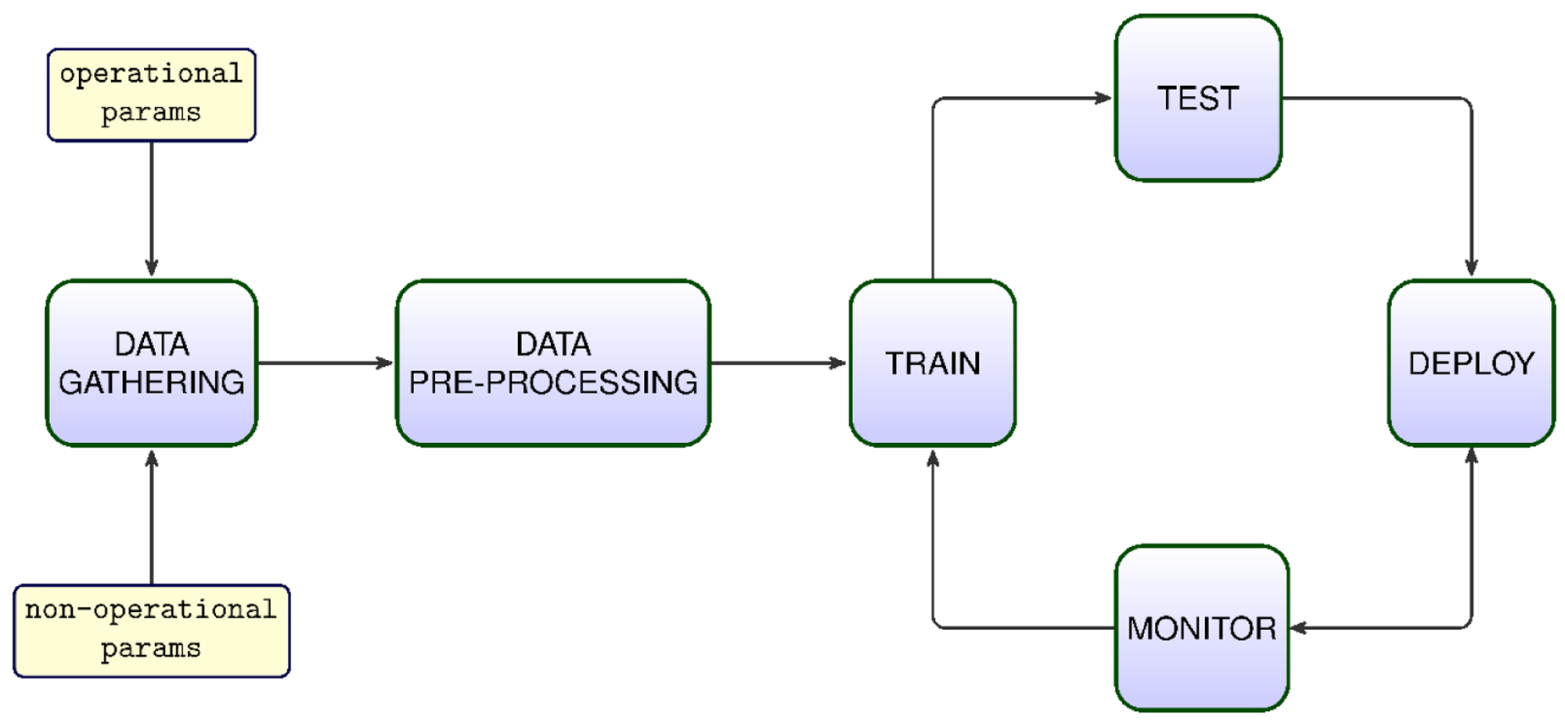
Experimental system as a small-scale mock-up of robotic inspection cell for quality control.

With the ever-increasing implementation of AI- and ML-based techniques in almost all engineering processes, these methods and approaches are prominent candidates for optimization, selection, decision-making and analyzing/monitoring steps in the V&V of autonomous systems. In the simulation environment for industrial robots ( Figure 13), several node points offer opportunities to conduct many of the following techniques: I) in safety trajectory optimization step with Moveit robot parameters, II) manipulation of sensor data in the Gazebo virtual simulation world, III) picture-to-picture translation with generative deep learning models for comparison and evaluation, IV) anomaly detection at component and system level by utilizing ML models, and V) monitoring and analyzing the overall system data. Moreover, the IMFIT fault-injection toolbox is included as a submodule whose main function is to present a set of ML tools to provide appropriate selections of fault injection types or instances in relation with the environmental conditions. This submodule acts together with the fault library, fault specification and source code scanner to employ insights for the fault injection planner.

Any V&V process based on ML or AI techniques follows a similar pipeline. This may be outlined as the steps of I) data gathering and storing, II) data preprocessing, III) training, IV) testing and optimization, and V) deployment and online integration. The data collection process is conducted in two modes of the real system: operational and non-operational. In the latter case, environmental parameters such as temperature and humidity are collected every 10 minutes. This frequency is increased to per minute in operational mode. The parameters related to the robotic arm, cameras, and other peripheral hardware are collected at a rate of 10 times/minute. Each collected data sample is written as a row on a CSV text file. Each sample is timestamped at the beginning of a row and a label associated with the current status is appended at the end of the row. The files are stored permanently. For an initial analysis, at least 100,000 rows of data samples are collected and stored. In the data preprocessing step, all columns (features) of the data are normalized in the range of [0, 1]. Any absent or missing information in any cell is filled with earlier adjacent data. Before any training or testing operation, fault types must be well-defined and the corresponding labels must be present in the data set, e.g. 0: non-fault, 1: sensor fault, 2: motor fault, 3: software fault, 4: PLC fault, 5: trajectory fault, 6: model fault. Another important issue about the data set is that it is divided into three subsets randomly: training (70%), validation (10%) and testing (20%). The validation subset is crucial in identifying any underfitting or overfitting issues about a model under test.

ML techniques fall into two broad categories: supervised and unsupervised. Before developing a classification or a regression analysis with a supervised method, some unsupervised techniques, such as the k-means clustering algorithm, may be utilized in order to extract any meaningful information about the dataset at present. Classification algorithms that are implemented and tested include support vector machines (SVM) and naive Bayes classifiers. Batch sizes and other hyperparameters are optimized during and after a training process by observing the process. Once satisfied within an acceptable accuracy rate, the parameters are recorded and documented for further inquiries. Test outputs are reported and analyzed in rich graphical representations ^76^. Any online inference infrastructure includes a dedicated server that is connected to the data source, the software bundle which runs for periodic classification and an alert system for an early warning mechanism. Virtualization with containers such as a Docker file is developed for easier installation of the bundle. These files may be utilized for both the training/testing or the deployment steps. ML-based key performance indicators (KPIs), e.g. testing to be completed for a single sample in less than 5 seconds, are observed during the lifecycle of the system under testing.

With the advent of the GPUs in scientific and industrial computing, deeper neural networks have been developed and become accessible during the last decade. Some of these deep learning models are combined with recurrent neural networks (RNN) and long-short term memory (LSTM) structures for temporal data analysis. The most successful outcomes for deep models have been achieved with images and videos lately due to wide utilization of convolutional neural networks (CNN). Classifiers, autoencoders, deep belief networks and generative models are a few examples of deep neural networks (DNN). Generative adversarial networks (GAN) ^77^ have gained attention lately in producing realistic images.

Quality control assessment of automotive body-in-white is based on 2D images. Presence-absence checking of the synthetic 2D images and the actual data obtained from the camera system is further extended to AI by developing and training GAN models. These models consist of two parts: a generator and a discriminator network. While the generator is trained to produce the most realistic image corresponding to a conditioning input image, the discriminator is forced to differentiate any generated image from a real one. This whole training process ends at an equilibrium where the generator and the discriminator no longer improve further.

## 5 Conclusion

This paper presents a use-case of the VALU3S project, namely " *an automated robot inspection cell for quality control of automotive body-in-white*". VALU3S aims to design, implement and evaluate state-of-the-art V&V methods and tools that reduce the time and cost needed to verify and validate automated systems concerning SCP requirements. The presented use case is original as it covers a very distinguished cyber-physical setting that is actively being used in an actual manufacturing process line in an automotive factory in Turkey. The quality inspection technique tackles realistic problems which are subject to serious implementation of the VALU3s V&V methodology. The vulnerabilities are covered holistically and the countermeasures against SCP threats are adaptable to similar cyber-physical settings.

The presented use-case is very suitable for demonstrating the project’s objectives since it is a highly complex, fully automatic, and challenging system, and prone to SCP threats by its nature. This paper presents the solution to tackling such SCP threats and covers the studies held within the first year of the project (since the beginning of May 2020 until April 2021). In this period, the project partners have determined the state of the art, V&V methods, workflows which are intended to develop, and the demonstration plans. The presented use case has the capacity to be generalized to other smart cyber-physical systems that can be revised for any robotic solutions in the Industry 4.0 context. The generalized approach consists of common tools and services which can be easily adapted to any cyber-physical setting where sensors, actuators and authorized persons interact with each other. For instance, the SCP countermeasures dealing with hardware- or software-based vulnerabilities, simulation techniques to model trajectory optimization, or AI-based techniques to detect SCP alarms can be applied to similar automated industrial settings with reasonable effort. In future phases of the project, the aim is to present the developed methods and workflows, including a demonstration of the given use case in future publications. Future studies will extend the scope to not only hardware-based cyberphysical protection at low level but also software-based techniques to tackle network intrusion or high-level attacks. In further steps, a wider portfolio of safety problems will be encountered by considering the safety trajectory optimization in fully automated and remote control settings, AI-based techniques will be improved for more effective and accurate anomaly detection,and the simulation of more SCP risks will be realized with a semantic threat model.

## Data availability

All data underlying the results are available as part of the article and no additional source data are required.
