# Supplementary material for: Verification and validation of an automated robot inspection cell for automotive body-in-white: a use case for the VALU3S ECSEL project
Source: Open Res Eur. 2021 Sep 29;1:115. [Version 1] doi: 10.12688/openreseurope.13627.1 (PMC10446043; doi:10.12688/openreseurope.13627.1)
Supplement: Peer review history

# Peer review: Reviewer response for version 1

**DOI:** 10.21956/openreseurope.14698.r27720

**Published:** 2021-10-25

**Authors:** Jaan Raik

**Affiliations:** 1 Talinn Tech. University, Tallinn, Estonia

This paper presents a Verification and Validation (V&V) approach used for the purpose of quality control of automotive body-in-white, proposed as one of the thirteen use cases in the Verification and Validation of Automated Systems’ Safety and Security (VALU3S) project which is funded under the Horizon 2020-ECSEL-2019-RIA (Electronic Components and Systems for European Leadership 2019 call for Research and Innovation Action) programme. The purpose of the use case illustrated in the paper is to demonstrate and evaluate an automated robot inspection cell for quality control of automotive body-in-white. The use case is active in a real automotive industrial setting in Otokar’s premises in Sakarya, Turkey. The joint study within this paper has been carried out by a consortium of three research-oriented small or medium enterprises (SMEs, Ergunler R&D Co., Inovasyon Co. and Techy Co.), a renowned university (Eskisehir Osmangazi University) and a distinguished large industrial organization in automotive sector (Otokar).

 The paper includes 5 sections. Section 1 provides a general summary of the paper. In Section 2, the VALU3S approach and its application within the context of the targeted use case is presented and the presented work is linked with ECSEL priority areas. The V&V methods, tools and concepts are presented with respect to the Safety, Cybersecurity and Privacy (SCP) requirements of the targeted industrial setting. Section 3 provides the related state-of-the-art regarding the fields of simulation-based fault injection, artificial intelligence/machine learning, safety trajectory optimization (formal verification) and cyber-physical security. Section 4 elaborates the targeted use case and presents the overall cyber-physical system and the underlying solutions to achieve the SCP requirements of a typical quality inspection system in automotive manufacturing.  Finally, Section 5 concludes the paper with provisional objectives and future work.

 The work is clearly and accurately presented. It provides a thorough background for the targeted use case, which is motivated and described in sufficient details. The included overview of related works to this topic cites relevant works from the fields related to the considered use case. There are sufficient details given for other practitioners to be able to apply the approach in their automated robot inspection use cases.

Is the case presented with sufficient detail to be useful for teaching or other practitioners?

Yes

Is the work clearly and accurately presented and does it cite the current literature?

Yes

If applicable, is the statistical analysis and its interpretation appropriate?

Not applicable

Are all the source data underlying the results available to ensure full reproducibility?

Yes

Are the conclusions drawn adequately supported by the results?

Yes

Is the background of the case’s history and progression described in sufficient detail?

Yes

Reviewer Expertise:

Dependability, verification and test of computing systems.

I confirm that I have read this submission and believe that I have an appropriate level of expertise to confirm that it is of an acceptable scientific standard.

# Peer review: Reviewer response for version 1

**DOI:** 10.21956/openreseurope.14698.r27719

**Published:** 2021-10-18

**Authors:** Baran Cürüklü

**Affiliations:** 1 Division of Intelligent Future Technologies, Malardalen University, Västerås, Sweden

This work stresses the importance of verification and validation (V&V) in the context of today's industrial applications. Furthermore, the paper assumes the results based on the ongoing ECSEL project VALU3S. The project VALU3S focuses on V&V for the requirements of safety, cybersecurity, and privacy (SCP). And this work is performed through 13 use cases. The project, thus this paper, has the potential of contributing to the work concerning quality inspection and control in Industry 4.0. The main context is the automotive industry, however, the rationale of the presented work is generic. However, the automotive industry is relevant in that there are increasing number of body parts, and the vehicles are becoming complex.

 The paper describes in detail one of the use cases in the project. In relation to the review questions above, there neither statistical data nor analysis. The explanation is clear and sound. It provides sufficient details for others to set up a similar systems. Supported by the many highly informative figures one would assume that this paper can be used in education.

Is the case presented with sufficient detail to be useful for teaching or other practitioners?

Yes

Is the work clearly and accurately presented and does it cite the current literature?

Yes

If applicable, is the statistical analysis and its interpretation appropriate?

Not applicable

Are all the source data underlying the results available to ensure full reproducibility?

Not applicable

Are the conclusions drawn adequately supported by the results?

Yes

Is the background of the case’s history and progression described in sufficient detail?

Yes

Reviewer Expertise:

Artificial intelligence, industrial robotics systems, multi-agent systems, cyber-physical systems

I confirm that I have read this submission and believe that I have an appropriate level of expertise to confirm that it is of an acceptable scientific standard.
